# Malignant Myopericytoma of Shoulder: A Rare Lesion

**DOI:** 10.21699/ajcr.v7i3.421

**Published:** 2016-06-15

**Authors:** Fariba Binesh, Reza Nafisi Moghadam, Masoud Shabani, Mohammad Reza Mortazavizadeh, Saeedeh Zare

**Affiliations:** 1 Department of Pathology, Shahid Sadoughi University of Medical Sciences, Yazd, Iran; 2 Department of Radiology, Shahid Sadoughi University of Medical Sciences, Yazd, Iran; 3 Department of Radiotherapy, Shahid Sadoughi University of Medical Sciences, Yazd, Iran; 4 Department of Oncology, Yazd Branch, Islamic Azad University, Yazd, Iran

**Keywords:** Myopericytoma, Malignant, Rare tumor

## Abstract

Myopericytoma is a soft tissue tumor with perivascular myoid differentiation. It accounts for 1% of the vascular tumors and involves mostly cutaneous or subcutaneous tissue of the limbs in adults. Malignant myopericytoma is exceedingly rare. A 15-year old girl presented with slowly progressive mass over left shoulder region. Histopathology and immunohistochemistry after complete excision revealed it as malignant myopericytoma.

## CASE REPORT

A 15-year-old girl presented to a local clinic with a slowly growing mass over the left shoulder for several months. Her past medical history was unremarkable. On physical examination, a 5 cm x 5 cm mass with relatively soft consistency was found in the left shoulder area. Its borders were relatively distinct and there was no fluctuation. Ultrasound revealed a well-demarcated heterogeneous solid mass (4.4 cm x 5.2 cm). The preoperative diagnosis was lipoma. MRI showed a soft tissue lobulated mass on the superior aspect of supraspinatus muscle (Fig. 1). Surgical excision of the lesion was performed. The mass was densely adherent to the surrounding structures and possibility of microscopic residual disease could not be ruled out. Histopathology showed a non-capsulated tumor composed of round to oval cells with eosinophilic cytoplasm, ill-defined cell borders, and myoid features with interspersed areas of closely packed small undifferentiated round cells. Broad zones of geographic pattern coagulative necrosis were seen. There was concentric perivascular accentuation of the cellular proliferation around hemangiopericytoma-like vascular spaces. A mitotic rate of up to 17 mitoses/10 high power fields was seen in the most cellular areas, including atypical mitosis. Immunohistochemically, most of tumor cells showed positivity for smooth muscle actin, vimentin, and CD99 but they were negative for cytokeratin, S100, desmin, and CD34 (Fig. 2). Based on these findings the diagnosis of malignant myopericytoma was made. Patient received six cycles of chemotherapy (ifosfamide-etoposide/cyclophosphamide-vincristine-doxorubicin) following which radiotherapy was given by using 18 MV photon of linear accelerator. A 54Gy radiation dose (in 30 fractions) was delivered. MRI examination performed at 18 months after surgery showed no evidence of any residual or recurrent tumor in the left shoulder region.

**Figure:**
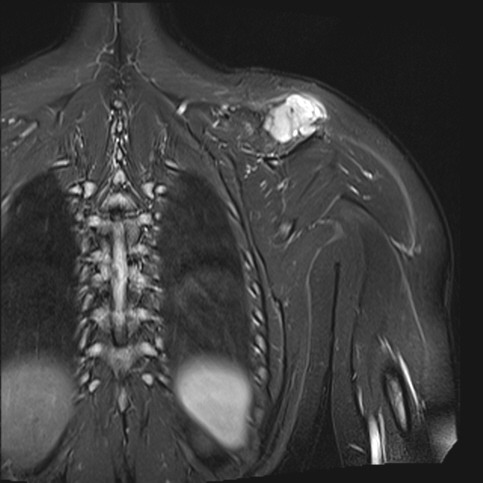
Figure 1:MRI revealed a soft tissue lobulated mass measuring 41 cm x 26 mm in the superior aspect of supraspinatus muscle.

**Figure:**
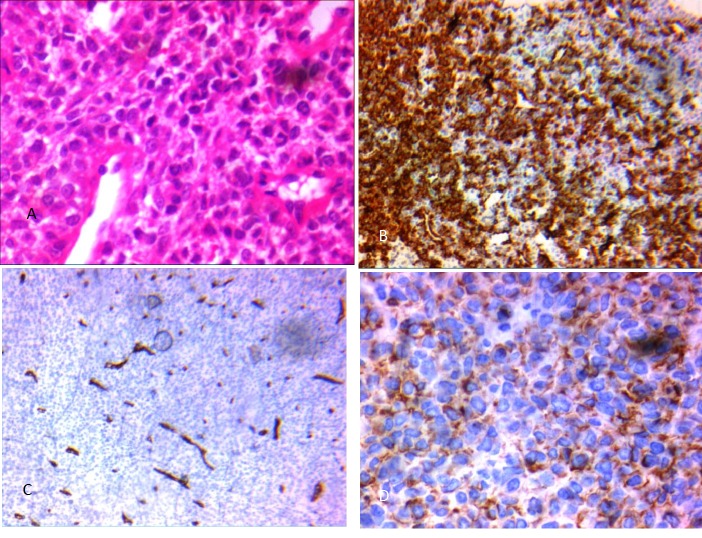
Figure 2:(A) Section show tumor tissue composed of round to oval cells with eosinophilic cytoplasm, ill-defined cell borders and myoid features (H and E stain X40). Tumor cells were positive for alpha smooth muscle actin (B) and CD99 (D) and negative for CD34 (C).

## DISCUSSION

Myopericytoma is an uncommon perivascular benign tumor having diameter often less than 2 cm. It develops as a single, painless, well circumscribed, and slowly growing lump in the subcutaneous tissues. Distal extremity is often involved however this tumor may occur in any anatomic site including neck, left atrium, urinary bladder, arm, thigh, and foot.[1-4] In the index case the left shoulder area was involved. These tumors are rarely reported in children. A slight male predominance is reported.[2]

Most myopericytomas are benign tumors; a malignant myopericytoma is extremely uncommon, often with local recurrence and distant metastases.[1,2] Microscopically, this tumor is composed of oval to fusiform cells which illustrate a notable concentric growth around the vessels. Malignancy is characterized by deeply infiltrative growth, severe atypia, increased mitotic index, high cellularity and necrosis.[1] All these findings were present on histopathology in our case. Immunohistochemistry also supported the diagnosis. It is frequently misdiagnosed as other sarcomas. Histologically, it has overlapping features with other perivascular tumors such as glomus tumor and myofibroma.[1]

These tumors require more extensive surgery with adjuvant therapy as malignant lesions may have an aggressive clinical course.[2] Radical surgical excision is the treatment of choice, as adjuvant chemotherapy and radiation therapy offer limited success in this malignancy. Our patient underwent surgery and received chemotherapy and radiotherapy. At 18 months follow-up, the general condition of the patient was good without any evidence of recurrence.

## Footnotes

**Source of Support:** Nil

**Conflict of Interest:** None declared

